# The Emerging Role of the Interplay Among Astrocytes, Microglia, and Neurons in the Hippocampus in Health and Disease

**DOI:** 10.3389/fnagi.2021.651973

**Published:** 2021-04-06

**Authors:** Daniele Lana, Filippo Ugolini, Daniele Nosi, Gary L. Wenk, Maria Grazia Giovannini

**Affiliations:** ^1^Clinical Pharmacology and Oncology, Department of Health Sciences, University of Florence, Florence, Italy; ^2^Section of Anatomopatology, Department of Health Sciences, University of Florence, Florence, Italy; ^3^Department of Experimental and Clinical Medicine, University of Florence, Florence, Italy; ^4^Department of Psychology, The Ohio State University, Columbus, OH, United States

**Keywords:** inflammaging, acute inflammation, CA1, CA3, triads, neurodegeneration, confocal microscopy

## Abstract

For over a century, neurons have been considered the basic functional units of the brain while glia only elements of support. Activation of glia has been long regarded detrimental for survival of neurons but more it appears that this is not the case in all circumstances. In this review, we report and discuss the recent literature on the alterations of astrocytes and microglia during inflammaging, the low-grade, slow, chronic inflammatory response that characterizes normal brain aging, and in acute inflammation. Becoming reactive, astrocytes and microglia undergo transcriptional, functional, and morphological changes that transform them into cells with different properties and functions, such as A1 and A2 astrocytes, and M1 and M2 microglia. This classification of microglia and astrocytes in two different, all-or-none states seems too simplistic, and does not correspond to the diverse variety of phenotypes so far found in the brain. Different interactions occur among the many cell populations of the central nervous system in health and disease conditions. Such interactions give rise to networks of morphological and functional reciprocal reliance and dependency. Alterations affecting one cell population reverberate to the others, favoring or dysregulating their activities. In the last part of this review, we present the modifications of the interplay between neurons and glia in rat models of brain aging and acute inflammation, focusing on the differences between CA1 and CA3 areas of the hippocampus, one of the brain regions most susceptible to different insults. With triple labeling fluorescent immunohistochemistry and confocal microscopy (TIC), it is possible to evaluate and compare quantitatively the morphological and functional alterations of the components of the neuron-astrocyte-microglia triad. In the contiguous and interconnected regions of rat hippocampus, CA1 and CA3 Stratum Radiatum, astrocytes and microglia show a different, finely regulated, and region-specific reactivity, demonstrating that glia responses vary in a significant manner from area to area. It will be of great interest to verify whether these differential reactivities of glia explain the diverse vulnerability of the hippocampal areas to aging or to different damaging insults, and particularly the higher sensitivity of CA1 pyramidal neurons to inflammatory stimuli.

## Introduction

“Hitherto, gentlemen, in considering the nervous system, I have only spoken of the really nervous parts of it. But if we would study the nervous system, it is extremely important to have a knowledge of that substance which lies between the proper nervous parts, holds them together and gives the whole its form in a greater or lesser degree” quoted from a lecture given by Rudolf Virchow (Charitè Hospital, Berlin, April 3rd, 1858).

For over a century, the brain was considered a network of neurons that communicate to each other in a vacuum filled by glia cells, solely with scaffolding and trophic roles. In the 1990s the scenario changed and astrocytes, microglia as well as oligodendrocytes started to be considered real partners of neurons and major players in the physiological and pathological conditions of the Central Nervous System (CNS). In the last few years, the neuron-centric view of the brain has changed toward a new perspective. Indeed, in the CNS glia subtypes, such as astrocytes, resident microglia, perivascular microglia, and oligodendrocytes outnumber neurons. Much effort is now devoted to understanding the cellular interplay and the molecular mechanisms that underlie the cellular responses that can bring to CNS repair after injury or to neurodegeneration.

The networks of cell intercommunication change during life of an organism, especially during aging or disease, and these modifications can have great consequences in the brain, and especially in the hippocampus. Indeed, it is becoming more and more evident that proper interplay among the main cells of the brain, neurons, astrocytes, and microglia is fundamental for the physiological and functional organization of the central nervous system. Recruitment and activation of astrocytes and microglia in a complex spatial and temporal pattern require well-organized intercommunication between neurons and glia as well as among glial cells.

While for a long time glia activation has been considered detrimental for survival of neurons, more recently intercommunication among astrocytes, microglia and neurons appears fundamental during brain development, for synaptogenesis, for maintenance of healthy synapses and for brain maturation (Pfrieger, 2009; Heneka et al., 2010). These interactions change not only in health and disease, but, in similar disease conditions, they are different in different regions of the brain, and can prevent, modulate or even control, but also exacerbate, the mechanisms of neurodegeneration. Activated astrocytes and microglia undergo a set of transcriptional changes that cause morphological and functional modifications, transforming them into cells with different functions and properties. Understanding in more details how and why glia engage in phenotypic switches and how all this can influence surrounding glia cells and neurons is of the utmost importance.

In this review, we will discuss, in the framework of the published literature, some findings from our laboratory that describe the involvement of the neurons, astrocytes, and microglia triad in rodent models of normal brain aging and acute brain inflammation. We used the method of the triple labeling fluorescent immunohistochemistry coupled to confocal microscopy (TIC) to evaluate and compare the morpho-functional alterations of the components of the neuron-astrocyte-microglia triad, focusing on the differences between CA1 and CA3 areas of the hippocampus in different experimental models of brain pathologies.

This understudied area of research has an emerging importance, and will undoubtedly teach us much about brain functions in physiological and pathological conditions.

## Different Responses of Astrocytes and Microglia in the Hippocampus During Aging or Acute Inflammation

As the expectancy of lifespan of the population in developed countries increases, age-dependent cognitive impairment represents a major challenge both for preclinical and clinical research. Impairment of cognitive functions together with a variety of neurobiological modifications characterizes brain aging. Inflammaging, the low-grade, slow, chronic upregulation of pro-inflammatory responses, represents the progressive neurobiological modification that occurs in the aging brain (Franceschi et al., 2007; Deleidi et al., 2015). Inflammaging hits the majority of the CNS, and particularly the hippocampus, a brain region involved in memory encoding, which during normal aging displays numerous electrophysiological, structural and morphological changes. These inflammaging-induced alterations may be the cause of memory loss typical of advanced age and of some neurodegenerative disorders. The CA1 and CA3 areas of the hippocampus cooperate in the mechanisms of short term memory encoding. The comparison between these two hippocampal areas is of fundamental importance, since they have important, although diverse, roles in memory encoding and since they undergo significant structural and morpho-functional modifications in AD and ischemia (Bartsch and Wulff, 2015). This comparison is also important because it can be the basis of the higher sensitivity of pyramidal neurons in CA1 to neurodegenerative injuries in both experimental animal models and in patients (Mueller et al., 2010; Small et al., 2011; Bartsch et al., 2015).

It is now becoming clear that the lack of microglial and/or astrocyte support seems to be responsible for neuronal degeneration rather than inflammation *per se*. Recent studies have demonstrated that at least two types of astrocytes and two types of microglia exist in the brain, with strikingly different properties. A1 and A2 astrocytes, and M1 and M2 microglia. A2 and M2 are non-reactive cells that seem to possess beneficial, neuroprotective properties. On the contrary, it appears that A1 and M1 are activated cells, with harmful properties for neurons. Indeed, A2 astrocytes promote outgrowth and survival of neurons, synaptogenesis, as well as phagocytosis, while A1 reactive astrocytes are neuroinflammatory, upregulate many genes increasing the expression of proinflammatory cytokines and other factors harmful for synapses (Liddelow and Barres, 2017). The M2 state of microglia is non-inflammatory and increases the secretion of a plethora of anti-inflammatory cytokines among which TNF-ß, IL-10, IL-4, and IL-13 (Allen and Barres, 2009). M1 microglia, activated by an acute insult, release proinflammatory cytokines among which TNFα, IL-6, IL-1, and IL-18 (Allen and Barres, 2009).

Nevertheless, according to Liddelow and Barres (2017) this quite recent classification of microglia and astrocytes in two different, all-or-none states appears rather too simplistic, and does not correspond to the variety of different phenotypes found in the CNS (De Biase et al., 2017; Keren-Shaul et al., 2017). A more recent hypothesis postulates that glia, as neurons, exist physiologically as heterogeneous, mixed populations, which may differ from a morphological and functional point of view. Consequently, there may exist *n* numbers of possible activation states of astrocytes and microglia, which are not related merely on the type of insult and the disease progression, but also on the cell type and on the CNS area in which cells are located (Zhang and Barres, 2010; Khakh and Sofroniew, 2015; Ben Haim and Rowitch, 2016; Liddelow and Barres, 2017; Khakh and Deneen, 2019; Pestana et al., 2020).

**Figure 1:**
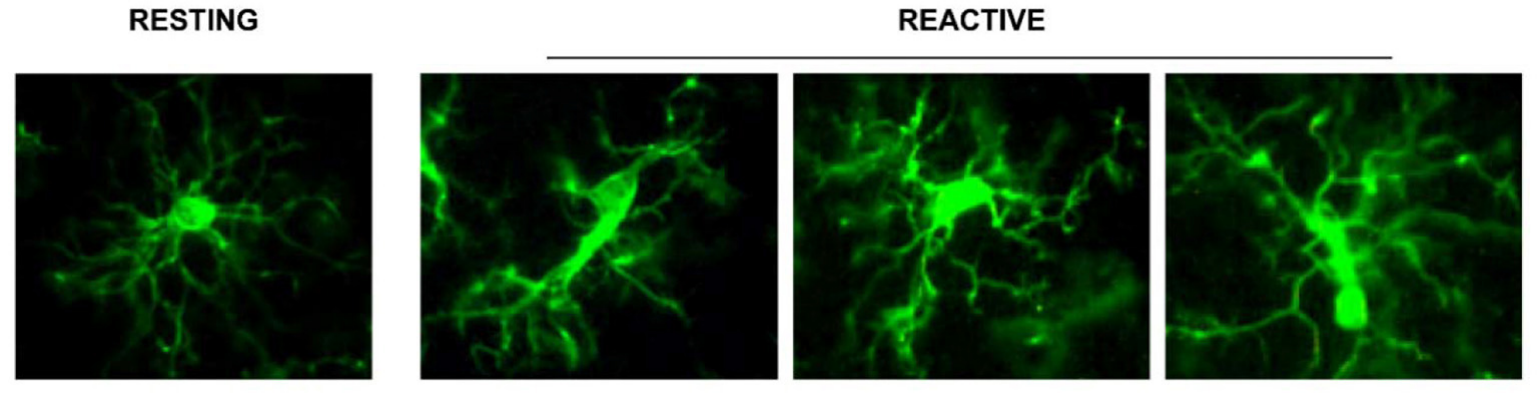
Representation of the different stages of microglia activation, marked with the anti IBA1 antibody [from Cerbai et al. (2012)].

Even just from a morphological point of view, in the aged rat hippocampus many types of microglia can be identified:

- branched microglia at rest (or quiescent microglia): CNS adult form, characterized by long and branched apophyses and a small cell body;
- non-phagocytic reactive microglia: intermediate stage between the branching and the phagocytic form with pleomorphic bi-or tri-polar cell body, or as spindle or rod-shaped cells
- phagocytic microglia: mainly of amoeboid shape and large dimensions. It is situated in brain areas affected by necrosis or inflammation; it phagocytes foreign materials and exposes immune-molecules for the activation of T-lymphocytes. Interacts also with astrocytes and neurons to quickly re-establish tissue homeostasis (Figure 1).

Therefore, activation profile of astrocytes and microglia can be imagined as a continuum rather than an all-or-none phenomenon. It is intriguing to determine whether astrocytes and microglia located in different brain areas react to a similar stimulus/insult with the same functional modifications (Martín-López et al., 2013; Bribian et al., 2018), or whether they react in a different way to a similar insult. In the first case, it is possible to envisage that they are controlled by intrinsic cues, independent from the environment; in the second one, it is possible that different signals derived from the environment diversify the cellular responses (Martín-López et al., 2013; Bribian et al., 2018). Thus, it appears that astrocyte and microglia activation expresses in an apparent continuum of intensities, and these effects are different in different areas of the brain. For instance, it has been demonstrated that astrocytes and microglia reactivities differ not only in aging and acute inflammation, but also between CA1 and CA3 hippocampus (Cerbai et al., 2012; Lana et al., 2016).

Furthermore, data from our and others' laboratories highlight the importance of the astrocyte-microglia dialogue in maintaining the homeostatic conditions that allow to maintain brain health. While adaptive astrogliosis has been shown to have beneficial effects for neurons, suppression of astrocytes activation may increase neuronal vulnerability, exacerbate the progression of pathological conditions and alter tissue regeneration (Sofroniew, 2009; Burda and Sofroniew, 2014; Pekny et al., 2014). Indeed, some data demonstrate that hypertrophy of astrocytes is not only a negative phenomenon, but in some cases may reflect adaptive plasticity of these cells, as demonstrated in aged rodents in which an enriched environment increases the morphological complexity of astrocytes (Rodríguez et al., 2013; Sampedro-Piquero et al., 2014).

The communication dialogue between astrocytes and microglia is a two-way street, mediated *via* their respective secretion molecules (Jha et al., 2019). Astrocytes release substances for the activation and proliferation of microglia, such as GM-CSF (Granulocyte-Macrophage Colony Stimulating Factors) and M-CSF (Macrophage Colony Stimulating Factors) (Watkins et al., 2007). Microglia communicate with astrocytes through the release of growth factors and cytokines, including IL-1, which regulates the proliferation of astrocytes (Streit and Xue, 2013). It is still considered valid that microglia cells are fundamental in establishing and maintaining inflammatory responses that may cause neurodegeneration (Glass et al., 2010). Nevertheless, it is now becoming clearer and clearer that they also actively maintain their protective role during normal aging (Faulkner et al., 2004; Myer et al., 2006; Hanisch and Kettenmann, 2007; Li et al., 2008) by disposing of dying cells (Block et al., 2007).

### Microglia in Acute Inflammation and Aging

Microglia cells have a defined territory and patrol the brain parenchyma by constantly moving their processes (Morsch et al., 2015) to detect and eliminate apoptotic neurons or neuronal debris or by phagocytosis or phagoptosis (Koenigsknecht-Talboo and Landreth, 2005). It has been shown that microglia cells have highly mobile, ramified processes that allow a continuous and dynamic patrolling of the healthy brain tissue. In this way, microglia have an active role in the surveillance of brain parenchyma to eliminate damaged neurons (Davalos et al., 2005; Nimmerjahn et al., 2005; Morsch et al., 2015). Activation of microglia is quick and leads to morphological, immunophenotypic and functional changes that stimulate the migration of microglia to the brain area affected by damage. The activated microglia, after having fulfilled its function of phagocytosis, is also able to regress rapidly to the quiescent form (Morsch et al., 2015).

It is becoming clear that activation of microglia is a highly regulated process. For instance, the transcripts of upregulated genes in microglia cells during aging-related chronic low-grade inflammation (inflammaging) differ fundamentally from those that are activated during acute inflammation, such as that induced by LPS infusion in young mice (Holtman et al., 2015). In acute inflammation, upregulated genes highly increase the expression of NF-κB signaling factors, whereas in inflammaging the signaling pathways related to phagosome, lysosome, or antigen presentation are upregulated, contributing to the phenotype of senescent microglia. In inflammaging, microglia display other specific morphological features in the hippocampus of aged rats (Cerbai et al., 2012) and in rodents express proinflammatory markers, such as MHC-II, IL-1β, IL-6, and TNFα, and contain lipofuscin granules [for references see Wolf et al. (2017)]. Aside from the differences in protein expression profile, in inflammaging both total and reactive microglia cells decrease in CA1 rat hippocampus (Cerbai et al., 2012), and their motility decreases in the retina of aged mice (Damani et al., 2011). Indeed, while in young animals microglia cells rapidly stretch their ramifications and increase their motility after administration of ATP, microglia cells during aging are much less mobile (Damani et al., 2011). In addition, microglia scavenging activity is different during aging and acute inflammation (Wolf et al., 2017; Lana et al., 2019) and the phagocytic activity and clearance capacity of microglia decreases with age, inversely correlating with Aβ plaque deposition in a mouse model of AD (Krabbe et al., 2013).

Activation of microglia, long considered only as a negative process that causes accumulation of neurotoxic phagocytes, is now considered a reversible, multi-staged process of generation of diverse types of reactive microglia cells that have protective capacity, at least in rodents (Hanisch and Kettenmann, 2007; Ransohoff and Perry, 2009; Kettenmann et al., 2013). Microglia ramifications are sensors that extend chemotactically toward injured cells in the “find-me” step of neuron phagocytosis (Hanisch and Kettenmann, 2007). Therefore, aging may impair and weaken the neuroprotective activity of microglia. Indeed, in rodents decreased migration of microglia may decrease its phagocytic efficacy, and favor the accumulation of degenerating neurons and proinflammatory neuronal toxic debris (Tian et al., 2012) a characteristic typical of CNS aging (Cerbai et al., 2012). Nevertheless, amplified, exaggerated, or chronic microglial activation can cause robust pathological changes and neurobehavioral complications, as in chronic inflammatory diseases (Glass et al., 2010; Norden and Godbout, 2013).

The endotoxin LPS, expressed on the wall of Gram-negative bacteria, behaves as an immunostimulant that activates microglia. The rapid activation of microglia after a damage, such as that caused by inoculation of LPS, is associated with the rapid activation of NF-kB, which does not require *de novo* protein expression. Indeed, the active form of NF-kB translocates from the cytoplasm to the nucleus, thus justifying the quick response of microglia to acute damage signals mediated by factors released by neurons, by pathogens or by immune cells. Inflammatory cytokines such as IL-6 and IL-1, both activators of NF-kB, recruit microglia (Gehrmann et al., 1995) while Interferon-γ and IL-4, released by T cells, stimulate the expression of MHC II on microglia, accelerating its proliferation. Matrix metalproteinases (MMPs), released from apoptotic cells, stimulate microglial activation. On the contrary, TGF-β1 and IL-10 negatively modulate microglia, reducing the expression of MHC II. Activated microglia rapidly express high levels of MHC II and several types of immunoglobulin family receptors, complement receptors, cytokines, chemokines (IFN-γ, IFN-β, IFN-α, IL-1, IL-6, IL-10, and IL-12) and receptors for mannose. Therefore, the cells acquire the ability to recognize and bind various antigens and present them to T lymphocytes (Rock et al., 2004).

Cytoskeleton remodeling of microglia can be monitored using immunostaining with antibodies for IBA1, a microglial marker of Ca^2+^-dependent actin polymerization. During aging, IBA1 immunostaining in microglia cells is significantly lower than in LPS rats, and microglia cells of aged rats are characterized by extremely limited branchings, which on the contrary are very ramified in LPS rats (Lana et al., 2019).

Microglia, with morphological characteristics of phagocytic cells, are significantly more numerous in acute inflammatory conditions than in inflammaging (Cerbai et al., 2012), mirroring a defense reaction of the adult CNS to the robust acute inflammatory stimulus. In acute inflammation in the rat, microglia survey the brain parenchyma, possibly to prevent spreading of proinflammatory, damaging molecules from apoptotic neurons and debris (Cerbai et al., 2012; Lana et al., 2016). This reactivity may be aimed at restoring normal physiological conditions, thus confirming the important role of microglia in the disposal of injured neurons in acute inflammation in the rat (Lana et al., 2019). The low-grade, chronic age-related processes of inflammaging may cause or be the cause of defective microglia patrolling of the parenchyma, and decreased targeting and phagocytosis of damaged neurons. Therefore, the efficiency of microglia in removal of damaged neurons from the nervous tissue may be lower during aging than in acute inflammatory conditions.

### Astrocytes in Acute Inflammation and Aging

**Figure 2:**
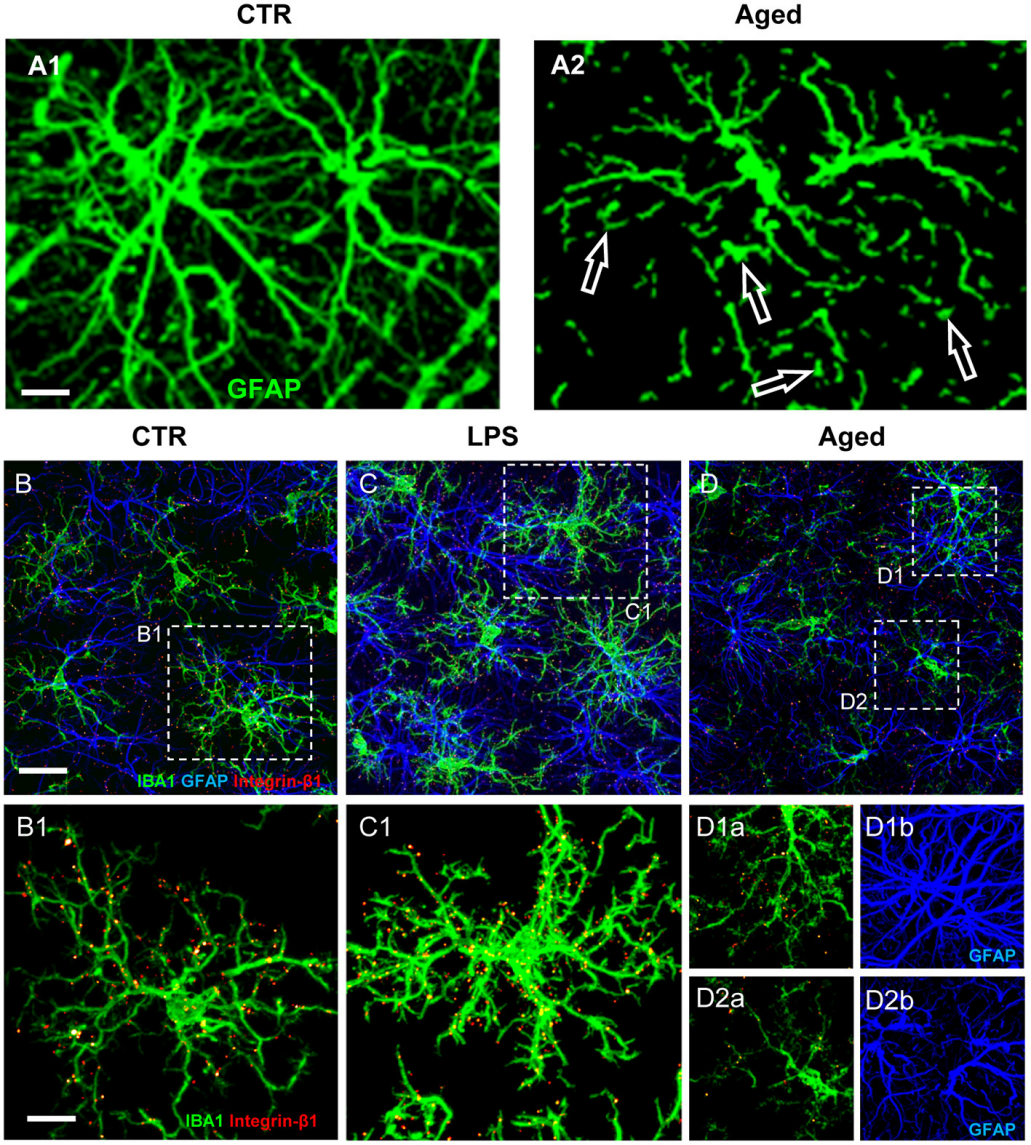
**(A)** Z-projections of confocal stacks acquired in CA1 hippocampus showing GFAP (green) immunostaining in control **(A1)** and aged **(A2)** rats. **(B–D)** Confocal acquisitions of IBA1 (green), GFAP (blue), and integrin-β1 (red) immunostaining in CA1 region of control **(B)**, LPS **(C)**, and aged **(D)** rats. **(B1–D2b)** Immunostaining of Integrin-β1 in microglia of control, LPS-treated and aged rats [magnifications of framed regions in **(B–D)**]; in **(B1,C1,D1b,D2b)** integrin-β1 colocalization on microglial cells is highlighted omitting GFAP immunostaining. Scale bars: **(A1,A2)**: 7.5 μm; **(B–D)**: 20 μm; **(B1–D2b)**: 7.5 μm [Modified from Lana et al., 2019)].

Understanding the multiple, contrasting roles of astrocytes in neuropathological mechanisms is of great interest to unravel the pathogenesis of many neurodegenerative disorders. Astrocytosis (Nichols et al., 1993; Morgan et al., 1997, 1999), defined as significant increase of GFAP expression, is present in the absence of astrocytes proliferation in the hippocampus of aged rats (Cerbai et al., 2012). Increased GFAP, possibly caused by increased transcription of the soluble fraction of GFAP in response to oxidative stress (Sohal and Weindruch, 1996; Morgan et al., 1997, 1999; Wu et al., 2005; Middeldorp and Hol, 2011; Clarke et al., 2018), in rodents is a feature of reactive/activated astrocytes (Zamanian et al., 2012; Burda and Sofroniew, 2014; Liddelow et al., 2017). These findings suggest that astrocytes become more reactive during aging, although not more numerous. Inflammaging causes loss of function in astrocytes, as well as in other cell types, reducing their ability to maintain a physiological, healthy environment (Palmer and Ousman, 2018). Inflammaging can negatively alter the mutual interactions of astrocytes with surrounding cells, being the cause and/or effect of the inflammatory state characteristic of aging. Healthy astrocytes are indispensable for synaptogenesis, for maintenance and maturation of healthy synapses (Pfrieger, 2009; Heneka et al., 2010), and contribute importantly to memory associated processes (Verkhratsky et al., 2011). In line with these housekeeping effects of astrocytes, in aged rats the density of astrocytes is lower in both CA1 and CA3 than in the corresponding areas of young rats (Cerbai et al., 2012; Lana et al., 2016). This finding is confirmed in the frontal and temporal white matter of aged subjects (Chen et al., 2016) and in the hippocampus of aged mice (Long et al., 1998; Mouton et al., 2002) or aged Brown Norway rats (Bhatnagari et al., 1997). Furthermore, the astrocytes in CA1 and CA3 of aged rats (Cerbai et al., 2012; Lana et al., 2016, 2019) show a different morphology and have shorter, highly fragmented branches (Figure 2A2, open arrows) compared to young rats astrocytes (Figure 2A1).

In healthy conditions the branches of astrocytes form a continuous functional syncytium (Kiyoshi and Zhou, 2019) that extends throughout the CA1 hippocampus (Figure 2A1), while during aging the syncytium is interrupted. These morphological modifications closely resemble the phenomenon of clasmatodendrosis, first evidenced by Alzheimer and successively named by Cajal (Penfield, 1928; Perez-Nievas and Serrano-Pozo, 2018; Tachibana et al., 2019). Clasmatodendrotic astrocytes, irreversibly injured by processes that cause energy failure and acidosis (Friede and van Houten, 1961), show morphofunctional modifications such as vacuolization and swelling of the cytoplasm as well as beading and disintegration of the branches (Tomimoto et al., 1997; Hulse et al., 2001).

Clasmatodendrosis, found in ischemic cardiovascular and Binswanger's diseases, characterized by dysfunction of the blood brain barrier (Tomimoto et al., 1997), as well as in Alzheimer's Disease, may represent a response of astrocytes to energy failure and mitochondrial inhibition (Friede and van Houten, 1961; Kraig and Chesler, 1990; Hulse et al., 2001). Indeed, some studies show that aged astrocytes are characterized by metabolic remodeling (Yin et al., 2014), and the oxidative metabolism in astrocytes increases with age, thus limiting their capability to supply neurons with metabolic substrates (Jiang and Cadenas, 2014). Typical hallmarks of brain aging are the impairment of energy metabolism and redox homeostasis, which also contribute to age-related neuronal degeneration and cognitive decline (Biessels and Kappelle, 2005; Boveris and Navarro, 2008). These modifications are even more evident at early stages of neurodegenerative disorders (Yin et al., 2014). Clasmatodendrosis is also caused *in vitro* by mild acidosis (Hulse et al., 2001), a microenvironmental condition present in the aging brain (Ross et al., 2010), in ischemia (Sahlas et al., 2002), and in Aβ-deposition (Su and Chang, 2001). Furthermore, in patients with severe head trauma, clasmatodendrosis is correlated to the presence of contusions or oedemas, and with significant decrease of survival (Sakai et al., 2013). The morphological modifications of astrocytes brought about by clasmatodendrosis have functional consequences. The shrunken arborization of astrocytes branches is indicative of the altered functions that astrocytes acquire during aging (Middeldorp and Hol, 2011), a condition in which astrocytes lose their housekeeping functions of scaffolding and trophic support for neurons and acquire a role in the disposal of neuronal debris (Cerbai et al., 2012; Lana et al., 2016). Clasmatodendrotic astrocytes lose their spatial orientation, thus resulting in reduced astrocytic syncytium and decreased coverage of synapses, and this modification can be a key factor in decreasing synaptic connectivity and neuronal homeostasis (Rodríguez et al., 2009; Verkhratsky et al., 2010). Clasmatodendrotic astrocytes have decreased endfeet coverage of brain vessels (Chen et al., 2016), compromising the blood brain barrier and the neurovascular unit, possibly contributing to vascular modifications observed during aging and at the early stages of AD (Bell and Zlokovic, 2009).

### Interplay of Microglia and Astrocytes in Aging and Acute Inflammation

As reported above, microglia cells survey the brain parenchyma to find and eliminate neuronal debris or apoptotic neurons by phagocytosis (Koenigsknecht-Talboo and Landreth, 2005). Focusing on the possible mutual mechanical interactions of microglia and astrocytes in CA1 of aged rats, evidences show that microglia are irregularly distributed and have reduced cytoplasmic projections (Cerbai et al., 2012; Wong, 2013; Deleidi et al., 2015; Lana et al., 2016). Decreased branchings and smaller volume of microglia projections are consistent with previous findings (Wong, 2013; von Bernhardi et al., 2015) and suggest that aged microglia have low efficient cytoskeletal remodeling of their projections and, consequently, decreased migration and phagocytic activity. When microglia cells are in the proximity of intact astrocytes, they display highly branched microglia projections (MPJs), establishing numerous contacts with astrocyte branches (APJs) (Figures 2B–D). At contact sites with astrocytes, microglia MPJs accumulate high levels of the mechanosensor Integrin-b1 (Lana et al., 2019) (Figures 2B1–D2). Integrin-b1 specifically localized in high density at the contacts MPJ/APJ, possibly influences the dynamic remodeling of MPJs (Lana et al., 2019), as also demonstrated in the spinal cord by Hara et al. (Hara et al., 2017). Mechanical stimuli up-regulate the expression of microglial Integrin-b1 (Milner et al., 2007), a membrane receptor involved in cell mechanosensing (Chen et al., 2017), thus promoting microglia branch extension (Ohsawa et al., 2010). In the aged rat hippocampus, microglia show irregular distribution and MPJs are smaller, shorter and have lower expression of IBA1 than those of young rats microglia (Cerbai et al., 2012; Wong, 2013; Deleidi et al., 2015; Lana et al., 2016). Microglia in the proximity of disrupted astrocyte branches, such as those of clasmatodendrotic astrocytes, show amoeboid morphology, have shorter and enlarged projections, and have low accumulation of Integrin-b1 (Lana et al., 2019). These data suggest that impairment of the direct interaction between astrocytes and microglia may hamper branching and migration of microglia. In acute inflammation, astrocytes form a continuum of APJs and highly ramified microglia that express increased levels of Integrin-b1, is in close contact with them (Figures 2C,C1). Increased expression of Integrin-b1 in microglia may correlate with an efficient microglia-astroglia interaction and ultimately with an efficient microglial targeting and phagocytosis of damaged neurons (Lana et al., 2019).

Nevertheless, it has also been demonstrated that, although microglia actively maintain their protective role during normal aging (Faulkner et al., 2004; Myer et al., 2006; Hanisch and Kettenmann, 2007; Li et al., 2008) by clearing dying neurons (Block et al., 2007), their capability is considerably impaired in an acute proinflammatory context. However, it should be underlined that the model of acute inflammation induced by LPS administration brings about different outcomes and activates different pathways in the diverse animal models used. For instance, in acute inflammatory conditions while activation of inducible NO synthase and expression of TLR4 are higher in mice than in human microglia, siglecs, molecules involved in innate immune responses, are more expresses in human than in mouse microglia (Butovsky et al., 2014). These differences may be responsible for different responses of microglia to noxious stimuli across species (Smith and Dragunow, 2014). In the zebrafish (*Danio rerio*), the scenario is still different since microglia is involved in the high capacity of recovery and regeneration of these animals after a noxious stimulus [for references see Var and Byrd-Jacobs (2020)]. Understanding the diverse microglia behavior in the different species may ultimately help to develop new methods for treating neuroinflammatory processes.

In the aged rat, microglia show impaired mobility and patrolling of brain parenchyma and their decreased phagocytic activity causes increased density of debris in CA1 and CA3 (Cerbai et al., 2012; Lana et al., 2016). Nevertheless, in accordance with previous findings (Koenigsknecht-Talboo and Landreth, 2005), although lesser in number, and less mobile, activated microglia in CA1 still actively scavenge apoptotic neurons, as demonstrated by phagocytosed neuronal debris present in their cytoplasm (Cerbai et al., 2012). On the contrary, under acute inflammatory conditions both microglia and astrocytes secrete Plasminogen activator inhibitor type 1 (PAI-1) that has an important role in microglia migration (Jeon et al., 2012) *via* the low-density lipoprotein receptor-related protein (LRP)-1/Janus kinase (JAK)/STAT1 axis and phagocytic activity *via* vitronectin and Toll-like receptor 2/6 (Jha et al., 2019). These data indicate that astrocytes play a regulatory role in migration and phagocytosis of microglia in an autocrine or paracrine manner.

In the mouse, microglia in normal aging up-regulates immune response signaling receptors (Grabert et al., 2016), neuroprotective signaling pathways (Hickman et al., 2013) and proinflammatory TNFα, IL-1α, and C1q (Liddelow et al., 2017), raising the question of whether these aging-induced modifications could promote the activation of astrocytes. The cytokines TNFα, IL-1α, and C1q are necessary and sufficient to induce the formation of A1 astrocytes, which, releasing a potent neurotoxin, are strongly neurotoxic and rapidly kill neurons (Liddelow et al., 2017). A1 astrocytes are less able to promote the formation of new synapses, causing a decrease in the excitatory function of CNS neurons. Other transcriptional and functional changes may occur in A1 astrocytes that contribute to cognitive decline in normal aging.

## Comparison Between CA1 and CA3

Neuroinflammation is generally believed to be characterized by activation of astroglia, typified by morphological changes, with low-moderate levels of inflammatory mediators in the parenchyma. It has been demonstrated that the responses of astrocytes and microglia to the same insults are diverse not only among different species and CNS areas, but also within the same areas of the hippocampus. The differential reactivity of astrocytes and microglia in normal hippocampus, during inflammaging and acute inflammation is reported in Table 1 (Cerbai et al., 2012; Lana et al., 2016). In the adult hippocampus, in basal conditions, microglia have much lower density in CA1 than in CA3. During aging, while astrocytes decrease significantly in all hippocampal subregions, total microglia decrease in CA1 and increase in CA3. In acute inflammation, both total and activated microglia increase in all hippocampal subregions, while astrocytes increase in CA3 only. It is of the utmost importance to understand whether the differences of astrocytes/microglia reactivity may explain the diverse susceptibility of the hippocampal areas to aging or to different inflammatory insults (Masgrau et al., 2017).

**Table 1:** Astrocytes and microglia responses to the same insult are not uniform, but in different stress conditions vary significantly from region to region.

		**CA1–SR**	**CA3–SR**
GFAP	Adult	522 ± 8	528 ± 27
	Aged	**−20%**	**−17%**
	LPS	+5 ns	**+21%**
IBA1	Adult	127 ± 12	239 ± 9
	Aged	**−26%**	**+16%**
	LPS	**+70%**	**+56%**
OX6	Adult	0.3 ± 0.2	4.5 ± 0.6
	Aged	**+3,060%**	**+1,160%**
	LPS	**+1,900%**	**+570%**

*Density of astrocytes (GFAP-positive), total microglia (IBA1-positive) and activated microglia (OX-6 positive) in SR of CA1 and CA3 of adult, aged and LPS-treated rats. Significant differences, expressed as percent of adult rats, are shown in bold. **CA1–SR:** from Cerbai et al., 2012; **CA3–SR**: from Lana et al., 2016*.

In CA1 area of the hippocampus of aged rats, microglia cells, although less numerous and less mobile than in young animals, maintain their scavenging activity, phagocytosing damaged, apoptotic neurons or debris. In CA1, fractalkine (CX3CL1) immunostaining is present on neurons actively phagocytosed by microglia (Cerbai et al., 2012). CX3CL1, a transmembrane glycoprotein located in neurons, forms by proteolysis cleavage a soluble chemokine domain that is released in the parenchyma. The two forms have different functions. Membrane bound CX3CL1 is an adhesion molecule, while its cleaved, soluble form, is a chemoattractant that recruits microglia that express its receptor (CX3CR1) to injured neurons (Harrison et al., 1998; Chapman et al., 2000; Cardona et al., 2006; Noda et al., 2011). Furthermore, the soluble form of CX3CL1 regulates microglia phagocytic activity (Cardona et al., 2006; Bhaskar et al., 2010; Lee et al., 2010; Liu et al., 2010). Since both microglia (Verge et al., 2004 and astrocytes (Dorf et al., 2000) express CX3CR1, fractalkine may be responsible for the recruitment of microglia and astrocytes in a well-organized topographic localization and spatial reciprocal interaction around apoptotic neurons to form triads. CX3CL1 contributes to the maintenance of microglia in a quiescent state (Lyons et al., 2009; Bachstetter et al., 2011). Nevertheless, soluble CX3CL1 increases in cerebral ischemia (Dénes et al., 2008), in response to glutamate stimulation (Chapman et al., 2000), during apoptosis (Fuller and Van Eldik, 2008), and is neuroprotective (Limatola et al., 2005) on cultured rat hippocampal neurons. CX3CL1 is also present in the spinal cord where, mediating the signaling between neurons and glia, it contributes to the insurgence of neuropathic pain (Clark and Malcangio, 2014). Thus, the effects of fractalkine may differ depending upon different stimuli, or upon the spatial localization of the cell (Lana et al., 2017a).

### Neurons Astrocytes Microglia Triads

A triad is defined as a cluster of cells in which a damaged/apoptotic neuron is surrounded by astrocytes that form a “microscar” around it, with the astrocyte branches that not only take contact with the neuronal cell membrane but also infiltrate the neuronal cell body (Cerbai et al., 2012; Lana et al., 2016). In the triad, a microglia cell is in the act of phagocytosing the neuronal cell body (Cerbai et al., 2012; Lana et al., 2016).

A1 astrocytes have been demonstrated *in vitro* to secrete a neurotoxin that induces neurons to undergo apoptosis (Liddelow and Barres, 2017; Liddelow et al., 2017). In addition, it has been demonstrated in a mouse model of Amyotrophic Lateral Sclerosis (ALS) that astrocytes release toxic factors that target specifically motor neurons and mediate cell death (Re et al., 2014). In a less neuronocentric view of neurodegenerative mechanisms, the alterations of astrocytes, in terms of number and morphology, with the consequential loss of their function, loss of maintenance of brain homeostasis, buffering of extracellular glutamate, as well as loss of supply of nutrients to neurons, may contribute to the spread of neuronal damage (Miller et al., 2017). During aging, astrocytes in the hippocampus and striatum of the mouse up-regulate a great number of reactive astrocytic genes (Clarke et al., 2018). The hippocampus and striatum, brain regions known to be most vulnerable in neurodegenerative diseases, are those where astrocytes upregulate A1 reactive genes the most (Burke and Barnes, 2006; Saxena and Caroni, 2011).

**Figure 3:**
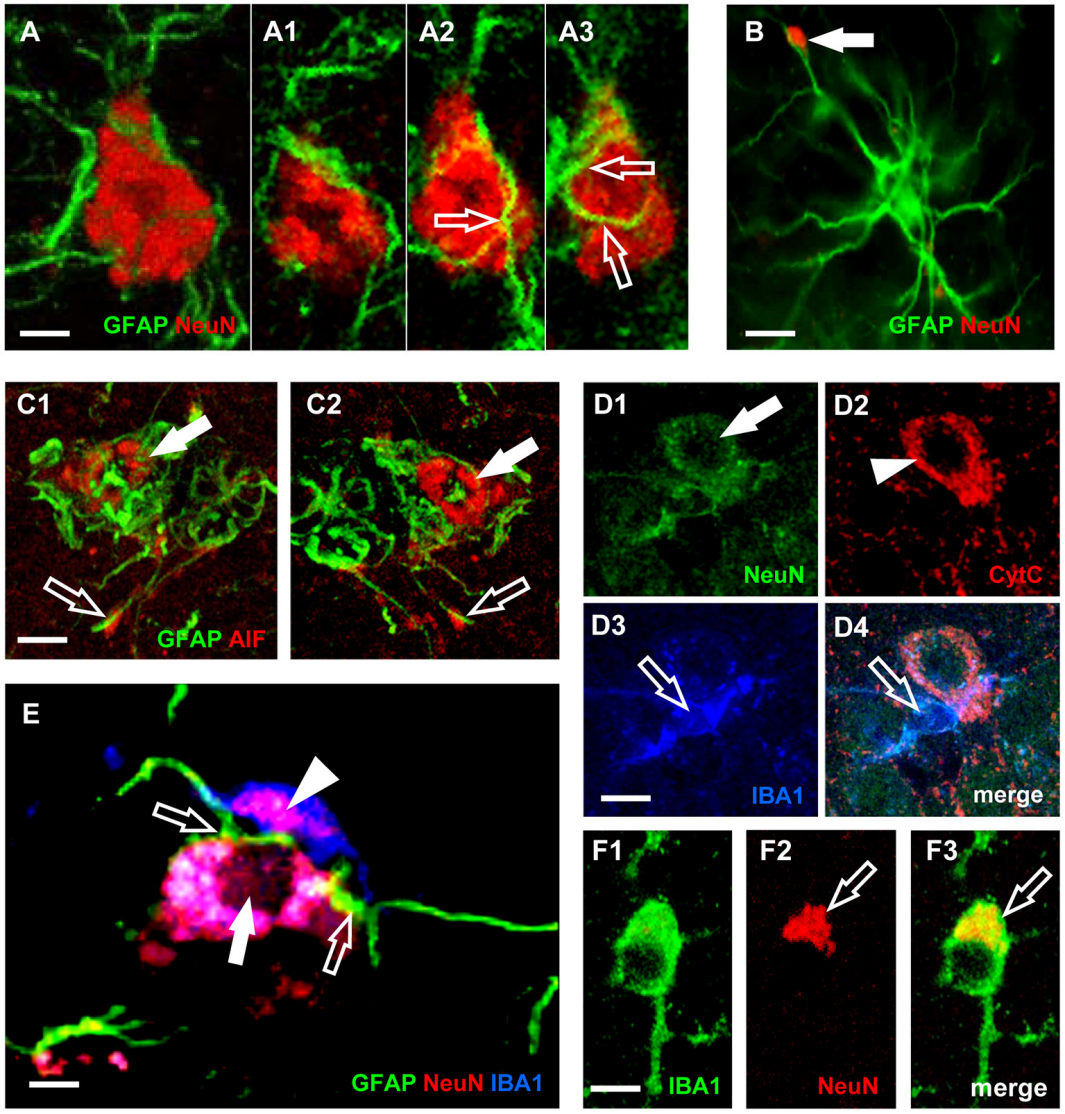
Characterization of neurons-astrocytes-microglia interplay and apoptosis. **(A)** Confocal microscopy acquisition of GFAP (green) and NeuN immunostaining (red). Scale bar: 5 μm **(A1–A3)**. Digital subslicing of the neuron in **(A)**. Images are the merge of two contiguous confocal acquisitions. Serial confocal images that digitally sub-slice the neuron cell body **(A1–A3)** clearly demonstrate that astrocyte branches are present inside and infiltrate the neuron body [open arrows in **(A2,A3)**]. Scale bar: 5 μm. **(B)** The arrow shows a neuronal debris closely apposed to an astrocyte branch. The presence in the neuron cytoplasm of AIF **(C1,C2)** or of diffuse CytC immunostaining **(D2)**, are clear signs of apoptosis (Suen et al., 2008). Scale bar: 15 μm. **(C1,C2)** Immunostaining for AIF (red) and GFAP (green). Scale bar: 5 μm. **(D1–D4)** Confocal acquisitions of NeuN (green), CytC (red), and IBA1 (blue) immunostaining acquired in CA3 region of aged rats. Digital subslice, total thickness 0.3 μm, and the merge of the three other images **(D4)**. Scale bar: 15 μm. **(E)** Digital subslicing of a neuron-astrocyte-microglia triad, obtained stacking seven contiguous confocal acquisitions. Open arrows point to astrocytes branches intermingling the body of a neuron. It is possible to appreciate the presence of an engulfed neuronal fragment within the body of the microglial cell (arrowhead). Scale bar: 10 μm. **(F1–F3)** Digital subslicing of the Str. Radiatum of an aged rat of a IBA1-positive microglia (green). It is possible to highlight the presence of a NeuN-positive neuronal fragment [red, **(F2)**] inside the microglial cell body [yellow-orange, **(F3)**, open arrow]. Scale bar: 7 μm [Modified from Cerbai et al. (2012), Lana et al. (2016)].

In line with these recent results, we had previously demonstrated in CA1, in CA3, as well as in DG of aged rats (Cerbai et al., 2012; Lana et al., 2016, 2017b) that astrocytes send branches to embrace, infiltrate and bisect apoptotic neurons (Figures 3C1,C2). This mechanism is finalized to the fragmentation of apoptotic neurons to form cellular debris that can be phagocytosed by microglia. This mechanism can protect and spare the neighboring cells from the damage caused by the release of proinflammatory products by the damaged neurons. Therefore, reactive astrogliosis can be beneficial, while suppression of astroglial reactivity may increase neuronal vulnerability, may exacerbate pathological development and may alter regeneration (Burda and Sofroniew, 2014; Pekny et al., 2014). However, when astrocyte reactivity becomes too intense, it is possible that the release of neurotoxic factors causes an intense neurotoxicity. Thus, astrocytes can behave as passive or active actors in causing or preventing neurodegeneration.

In the triads, astrocytes branches infiltrate and bisect the apoptotic neuron cytoplasm, which is phagocytosed by microglia as shown in Figure 3A. Neurons infiltrated by astrocyte branches show signs of degeneration, such as karyorrexhis (Figures 3E,D1), and apoptosis (Figures 3C1,C2,D2). Apoptotic neurons are in close contact with microglia cells that phagocytose the neuron cell body or cellular debris (Figures 3D3,D4,F1–F3, open arrows).

Both microglia and astrocytes can recognize danger signals in the parenchyma, including those released by cellular debris produced from apoptotic cells. Therefore, it appears that microglia and astrocytes can cooperate to help clearing apoptotic neurons or neuronal debris (Medzhitov and Janeway, 2002; Milligan and Watkins, 2009) in a concerted effort to prevent or reduce the release of proinflammatory mediators and damage to neighboring neurons (Nguyen et al., 2002; Turrin and Rivest, 2006). Nevertheless, examples exist of positive effects of active microglia and production of cytokines in early brain development (Salter and Beggs, 2014), in synaptic pruning (Schafer and Stevens, 2013) and in learning and memory mechanisms (Ziv et al., 2006; Derecki et al., 2010).

During normal aging, apoptosis is a physiological mechanism that maintains normal tissue homeostasis through resolution of low-grade inflammation (Gupta et al., 2006). As a consequence, under conditions of physiological aging, the effects of astrocytes and microglia are protective, removing neuronal debris by phagocytosis or entire neurons by phagoptosis (Lana et al., 2017a), pruning dysfunctional synapses and controlling inflammation and the diffusion of cellular damage.

In the hippocampus of aged rats, CA1 and CA3 pyramidal neurons significantly decrease (Cerbai et al., 2012; Osborn et al., 2016), possibly because of increased disposal of degenerating neurons paralleled by decreased neurogenesis (Kuhn et al., 1996) during low-grade but long-lasting inflammaging. In acute inflammation caused by LPS, the neurons dying for apoptotic mechanisms can be replaced by new neurons generated by neurogenesis. Indeed, GFAP/vimentin KO increases cellular proliferation and neurogenesis in the granular layer of the dentate gyrus (Larsson et al., 2004). Furthermore, GFAP null astrocytes support neuronal survival and outgrowth of neurites better than their wild-type counterparts (Menet et al., 2000), indicating that aging of astrocytes associated with increased expression of GFAP can repress the capacity of astrocytes to increase neurogenesis and neuronal protection in the brain. Increased neuron degeneration by aging, paralleled by the age-related decrease of neurogenesis, which decreases physiological neuron replacement, may thus be one of the causes of the age-related decrease of neurons, and may contribute to aging dependent impairments of brain function.

Active and controlled cell death may serve a homeostatic function in regulating the number of cell population in healthy and pathological conditions (Kerr et al., 1972; Becker and Bonni, 2004). How apoptosis causes neurons to be disposed of is still a matter of debate. One of the main mechanism is the release of intercellular signals from neurons which induce phagocytic cells such as microglia to engulf and consume the neuron (Noda et al., 2011; Cerbai et al., 2012). Astrocytes and microglia express receptors able to recognize molecules released by neurons (Harrison et al., 1998; Noda et al., 2011), inducing the phagocytosis of damaged cells or neuronal debris. Therefore, triad formation seems a specific mechanism for clearance of degenerating neurons, through phagocytosis, or through phagoptosis (Brown and Neher, 2012; Fricker et al., 2012). Phagoptosis is triggered by cell stress, which is too mild to cause the death of the cell, too serious to allow readaptation of the cell from the damage, and sufficiently strong to recruit astrocytes and microglia for phagocytosis (Kao et al., 2011). During aging, although microglia increase in number, the cells have morphological modifications that cause less neuroprotective and defensive capabilities (Streit et al., 2009; Tremblay et al., 2011; Streit and Xue, 2013). Thus, targeting the triads may represent a therapeutic strategy, which may control release of proinflammatory signals from damaged neurons and spread of further cellular damage to neighboring cells.

In normal brain aging and acute neuroinflammation caused by LPS administration in the rat (Cerbai et al., 2012; Lana et al., 2016), many neurons in the Str. Radiatum of hippocampal areas CA1 and CA3 are apoptotic pyramidal neurons that form triads with astrocytes and microglia. They show increased activation of p38MAPK (Xia et al., 1995) and increased immunostaining for CytC (Ow et al., 2008) and AIF (Lorenzo et al., 1999). Some of the apoptotic neurons in the aged rats, located very close to the Str. Pyramidalis but clearly detached from it, are surrounded and infiltrated by astrocyte branches, possibly recruited by overexpression of Connexin43 (Cx43) (Cerbai et al., 2012). Astrocyte branches embrace, infiltrate and wedge the apoptotic neuron and form debris. It appears that the astrocyte branches themselves remove the damaged, apoptotic neuron from the Str. Pyramidalis, signaling via intercellular molecules such as the Cx43 and/or CX3CL1 (Noda et al., 2011). Indeed, it is known that during the first steps of apoptosis caspases break the cell cytoskeleton, allowing the apoptotic cell to detach from the surrounding, healthy cells (Böhm, 2003). This mechanism may explain how pyramidalis apoptotic neurons migrate from the Str. Pyramidalis to the Str. Radiatum to form triads in which phagocytosis may take place. Apoptosis, active and controlled cell death, may serve a homeostatic function, regulating the number of cells, not only in pathological conditions but also in the healthy brain (Becker and Bonni, 2004). For instance, the role of microglia is critical in the early stages of embryonic development in which excess neurons are produced; later, these neurons face programmed cell death and break up into small apoptotic bodies, removed by microglia (Pont-Lezica et al., 2011). Microglia change their morphology in relation to the development of the CNS and in particular pathological conditions.

We first demonstrated in the hippocampus of aged rats that in the triads, astrocyte branches infiltrate a damaged, apoptotic neuron, to bisect the dying neuron and form neuronal debris (Cerbai et al., 2012) which are significantly numerous in aged and LPS-treated rats. Neuronal debris are all closely apposed to astrocyte branches and are phagocytosed by microglia. Therefore, it appears that both microglia and astrocytes can recognize danger signals in the surrounding parenchyma and can interact and help clearing apoptotic cells and the resulting debris (Medzhitov and Janeway, 2002; Milligan and Watkins, 2009), at least in rodents.

Nevertheless, in the contiguous and interconnected CA1 and CA3 subregions of rat hippocampus, astrocytes and microglia show very different reactivity, as reported in Table 1 (Cerbai et al., 2012; Lana et al., 2016), demonstrating that the responses of astrocytes and microglial to the same insult are not uniform but vary significantly from area to area and in different conditions. It was demonstrated in the rat that activated microglia are seen diffusely scattered throughout the brain after 2 days of LPS infusion (Wenk and Barnes, 2000). During the next weeks, the number of activated microglia gradually decreases in all cerebral regions and later the greatest inflammatory response is concentrated within the hippocampus (Wenk and Barnes, 2000). These findings suggest that in the rat LPS initiates a cascade of biochemical processes that show time dependent, regional and cell specific changes that are maximal after 4 weeks of LPS infusion (Hauss-Wegrzyniak et al., 1998; Wenk and Barnes, 2000).

**Figure 4:**
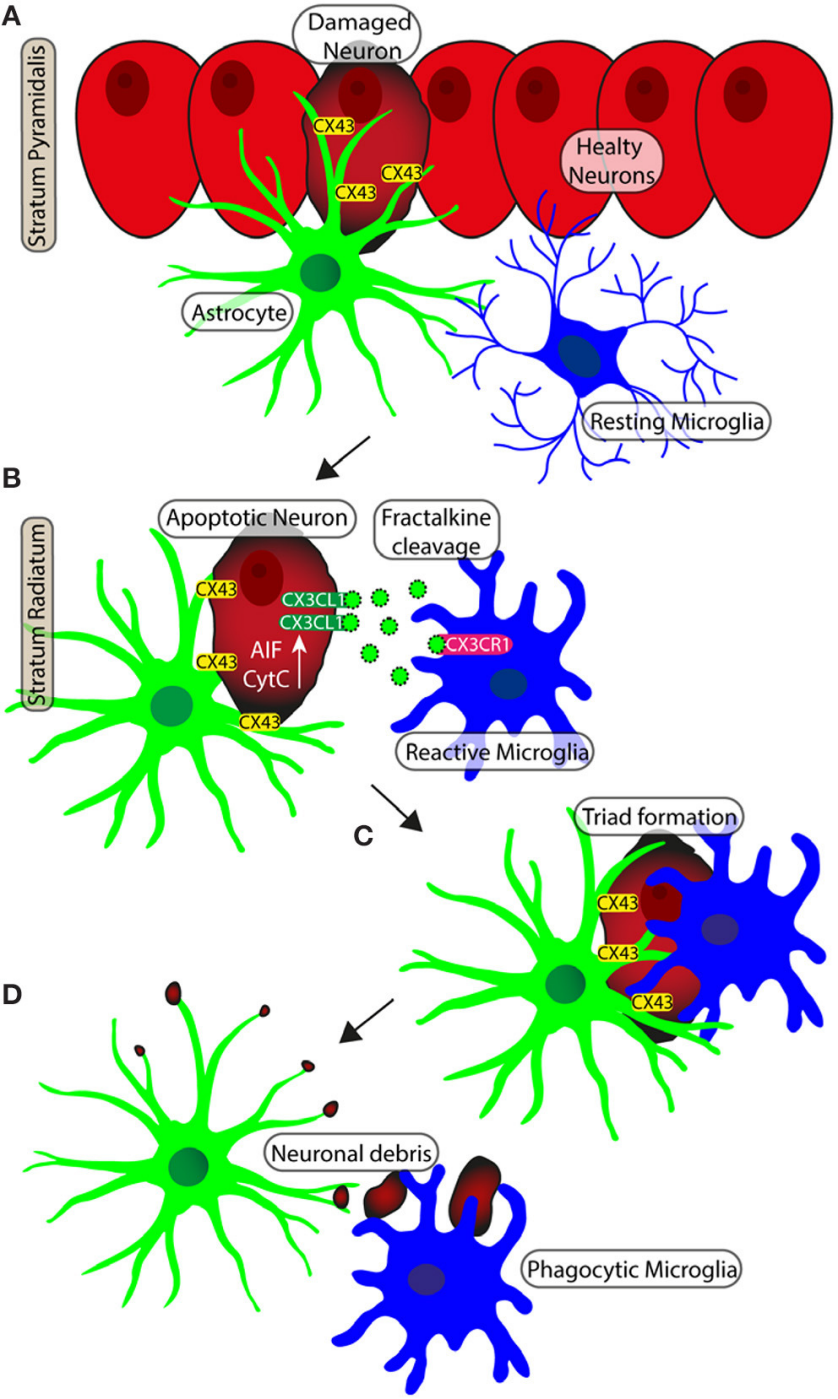
Schematic representation of neuron-astrocytes-microglia interplay during neurodegeneration. **(A)** Apoptotic neurons of pyramidal layer show diffuse cytoplasmic staining of CytC or AIF. Gap junctions (Connexine 43) mediate the recruitment of astrocytes on neurons. **(B)** The apoptotic neuron is detached from the Pyramidal layer to becoming an ectopic pyramidal neuron. CX3CL1 (fraktalkine) is released by the ectopic neuron and the astrocyte branches take close contact with the neuron. **(C)** Microglia are recruited and astrocytes branches infiltrate the body of the damaged neuron. **(D)** The damaged neuron is bisected and disgregated in cell debris. Phagocytic microglia mediate the clearance of cell debris.

Rapid neuroprotective effect of microglia is facilitated by its regular distribution in the tissue, which minimizes the distance from possible pro-inflammatory triggers, such as cell debris and entire damaged neurons. Astrocytes constantly contact neuron surfaces, and interact with neuron debris (Reemst et al., 2016). Therefore, the contiguous meshwork of their projections may be the first structures that enter in contact with possible targets of microglia phagocytosis. We demonstrated that direct cell-cell interactions exist between astrocytes and microglia, which can influence and mediate microglial branching, addressing branch tree extension toward pro-inflammatory triggers. The disruption of astrocyte meshwork that occurs during aging, could be related to dysregulation of microglial defensive activity (Lana et al., 2019). A schematic representation of neuron-astrocytes microglia interplay in the triads is shown in Figure 4.

The chronic, sterile, low-grade neuroinflammation that develops during normal brain aging (Franceschi et al., 2018) eventually activates microglia and astrocytes that first cooperate to maintain brain homeostasis. We now know a degree of neuroinflammatory cytokines that act on other cells to influence cellular biochemistry, physiology and development that can represent or not pathological neuroinflammation, but rather demonstrate a way in which signals are communicated within the CNS. When the inflammation becomes more intense, microglia and astrocytes start releasing proinflammatory and neurotoxic mediators and increase the expression of inflammation-related proteins (Liddelow and Barres, 2017). All these mechanisms start a vicious circle that causes progressive impaired interplay between neurons, astrocytes and microglia that, when too intense, may be responsible for derangements from normal brain aging to neurodegenerative diseases (De Keyser et al., 2008; Sofroniew, 2009).

## Conclusions

The data reported in the present review expand the wealthy panel of interactions that occur among the different cell populations of the CNS and add plausibility to the idea that such interactions bring about a network of morphological and functional reciprocal reliance and dependency. To comprehend the peculiar aspects of the onset and progression of neuroinflammation, it is necessary to understand and take into consideration that any tissue, and mainly the nervous tissue, is a mere collection of single elements but rather by interacting and interdependent cell populations that cooperate to maintain the homeostasis and functionality of the organ. Different types of alterations affecting one population reasonably reverberate to the others either favoring or dysregulating their activities.

It should be pointed out, however, that the majority of the studies reported in this review were performed in rat or mouse experimental models. Many studies indicate that rodent and human microglia and astrocytes react differently to mediators of neuroinflammation, have a different rate of proliferation or activation and express different proteins and enzymes in response to external stimuli such as proinflammatory mediators (Smith and Dragunow, 2014; Streit et al., 2014; Wolf et al., 2017). Not only animal models but also those performed in specimens from human tissue, as all models, have positive and negative aspects. It is of the utmost importance, however, to understand that this experimental field, that dates over 150 years, is still in its infancy.

## Author Contributions

MG and DL wrote the paper. FU and DN contributed to the preparation of the figures. MG and GW edited and revised the manuscript. All authors read and approved the final manuscript.

## Conflict of Interest

The authors declare that the research was conducted in the absence of any commercial or financial relationships that could be construed as a potential conflict of interest.

## Abbreviations

AD: Alzheimer's Disease
AIF: Apoptosis Inducing Factor
ALS: Amyotrophic Lateral Sclerosis
APJs: Astrocytes branches
CNS: Central Nervous System
Cx43: Connexin43
CytC: Cytochrome C
GFAP: Glial Fibrillary Acidic Protein
GM-CSF: Granulocyte Macrophage-Colony Stimulating Factors
IBA1: Ionized calcium Binding Adaptor molecule
IFN: Interferon
IL: Interleukine
JAK: Janus kinase
LPS: Lipopolysaccharide
LRP-1: Lipoprotein receptor-related protein
M-CSF: Macrophage-Colony Stimulating Factors
MAPK: Mitogen Activated Protein Kinase
MHC: Major Histocompatibility Complex
MMP: Matrix metalproteinases
MPJs: Microglia projections
MRI: Magnetic Resonance Imaging
MPM: Multiphoton Microscopy
NF-Kb: Nuclear Factor-kappa B
PAI-1: Plasminogen activator inhibitor type 1
PET: Positron Emission Tomography
SPECT: Positron Emission Computed Tomography
SR: Str. Radiatum
TGF-β: Transforming Growth Factor-beta
TIC: Triple labeling fluorescent Immunohistochemistry coupled to Confocal microscopy
TLR4: Toll-like receptor 4
TNF: Tumor Necrosis Factor.
